# The complete mitochondrial genome of *Bufotes zamdaensis* (Anura: Bufonidae)

**DOI:** 10.1080/23802359.2019.1624209

**Published:** 2019-07-10

**Authors:** Shuai Li, Li-Fang Peng, Shun-Qing Lu, Song Huang

**Affiliations:** College of Life and Environment Sciences, Huangshan University, Huangshan, China

**Keywords:** Mitogenome, *Bufotes zamdaensis*, phylogenetic analysis

## Abstract

The complete mitochondrial genome sequence of *Bufotes zamdaensis* was reported in the present research by shotgun sequencing. The total length of mitogenome is 17,189 bp, and contains 13 protein-coding genes, 22 tRNA genes, two ribosome RNA genes, and one D-loop region. All gene conformations were consistent with the gene arrangement in vertebrates. Most of the genes of *B*. *zamdaensis* were distributed on the H-strand, except for the ND6 subunit gene and eight tRNA genes which were encoded on the L-strand. Phylogenetic analysis of *B*. *zamdaensis* and 12 other closely amphibian species was reconstructed using Maximum-likelihood methods. The sequences of *B*. *zamdaensis* were clustered in genus *Bufotes*. The phylogenetic analyses based on these mitogenomes presented here will be useful to further insights on the evolutionary relationships of Bufonidae.

The genus *Bufotes* is a monophyletic clade in the family Bufonidae (Pyron and Wiens 2011) which contains 14 known species. There is only two species *B*. *zamdaensis* and *B.* *pewzowi* distributed in China (Frost 2016). *Bufotes zamdaensis* (Fei et al. 1999) is currently distributed in Zanda County, Ngari Prefecture, Tibetan Autonomous Region, China (Fei et al. 2012). This species is a mountain marsh grass or near the pond species, which inhabits about 2900 m sea level (Fei et al. 1999). In this research, we determined and described the mitogenome sequence of *B*. *zamdaensis* in order to obtain basic genetic information about this species.

The specimen of *B*. *zamdaensis* (Voucher numbers: HS17502) was collected from Diya township, Zanda County, Ngari Prefecture, Tibetan Autonomous Region, China on 3 July 2017. It was preserved and deposited in the Museum of Huangshan University. Total genomic DNA was extracted from the liver tissue of this specimen using Takara MiniBEST Universal Genomic DNA Extraction Kit (Takara, Dalian, China). The complete mitochondrial genome sequence in was reported in this research by shotgun sequencing.

The positions of RNA genes were predicted using the MITOS (Bernt et al. 2013), and the locations of protein-coding genes were identified by comparing with the homologous genes of other related species. The complete mitogenome sequence of *B*. *zamdaensis* is a closed circular molecule composed of 17,189 bp, which has been submitted to GenBank with accession number is MK867845, containing 13 typical vertebrate protein-coding genes (PCGs), 22 transfer RNA (tRNA) genes, two ribosomal RNA (rRNA) genes, and one control region (D-loop). The base composition was 34.3% for A, 24.8% for T, 12.6% for G, and 28.2% for C. Among the mitochondrial protein coding genes, the ATP8 was the shortest, whereas the ND5 was the longest. Most of the *B*. *zamdaensis* mitochondrial genes are encoded on the H-strand except for the ND6 gene and eight tRNA genes, which are encoded on the L-strand. The gene order, contents and base composition are identical to those found in typical vertebrates (Boore 1999; Sorenson et al. 1999).

In order to convince the mitochondrial DNA sequences obtained in this study, we used the 13 PCG genes of the mitogenome of *B*. *zamdaensis* and other 12 closely related species to construct the phylogenetic tree using Maximum-likelihood (ML) methods in http://www.phylo.org/portal2/login!input.action. The phylogenetic tree (ML Tree) indicated that the mitogenome of this species was genetically closest to that of *B*. *raddei* (Figure 1) with a highly support, which is in accordance with the current morphological classification. It indicated that our new determined mitogenome sequences could meet the demands and explain some evolution issues.

**Figure 1.**
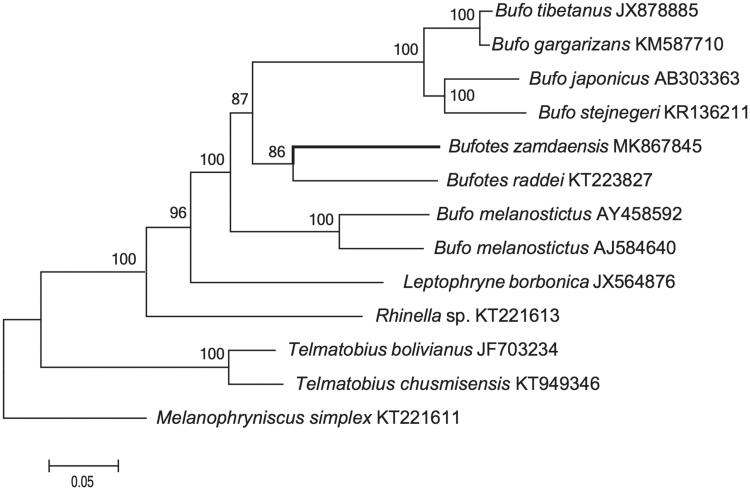
A maximum-likelihood (ML) tree of *Bufotes zamdaensis* in this study and 12 related species was constructed based on the dataset of the whole mitochondrial genome by online tool RAxML. The numbers above the branch meant bootstrap value. Bold black branches highlighted the study species and corresponding phylogenetic classification.
